# Validation of the Dynamic Direct Exposure Method for Toxicity Testing of Diesel Exhaust *In Vitro*

**DOI:** 10.1155/2013/139512

**Published:** 2013-08-05

**Authors:** Lucky Joeng, Amanda Hayes, Shahnaz Bakand

**Affiliations:** ^1^Chemical Safety and Applied Toxicology (CSAT) Laboratories, School of Risk and Safety Sciences, The University of New South Wales (UNSW), Sydney, NSW 2052, Australia; ^2^School of Chemistry, The University of New South Wales (UNSW), Sydney, NSW 2052, Australia; ^3^Department of Occupational Health, School of Public Health, Iran University of Medical Sciences, Tehran, Iran

## Abstract

Diesel exhaust emission is a major health concern because of the complex nature of its gaseous content (e.g., NO_2_, NO, CO, and CO_2_) and high concentration of particulate matter (PM) less than 2.5 **μ**m which allows for deeper penetration into the human pulmonary system upon inhalation. The aim of this research was to elucidate the potential toxic effects of diesel exhaust on a human pulmonary-based cellular system. Validation of a dynamic direct exposure method for both laboratory (230 hp Volvo truck engine) and field (Volkswagen Passat passenger car) diesel engines, at idle mode, was implemented. Human pulmonary type II epithelial cells (A549) grown on porous membranes were exposed to unmodified diesel exhaust at a low flow rate (37.5 mL/min). In parallel, diesel emission sampling was also conducted using real-time air monitoring techniques. Induced cellular effects were assessed using a range of *in vitro* cytotoxicity assays (MTS, ATP, and NRU). Reduction of cell viability was observed in a time-dependent manner following 30–60 mins of exposure with NRU as the most sensitive assay. The results suggest that the dynamic direct exposure method has the potential to be implemented for both laboratory- and field-based *in vitro* toxicity studies of diesel exhaust emissions.

## 1. Introduction

Air pollution is a major concern to human health. Epidemiological studies have shown that air pollution poses the greatest human health risks to the young and elderly and those with chronic cardiovascular disease, asthma, or influenza [1–6]. Air pollution is most prevalent in largely populated urban areas such as cities with high-density traffic that consequently poses a higher risk of adverse health effects.

Mobile sources such as motor vehicles are the main contributor to urban air pollution, emitting gases, particulates, and/or mixtures of these into the atmosphere. Motor vehicles such as diesel powered passenger vehicles are gaining popularity over the traditional petrol engines as diesel engines have higher fuel efficiency due to the more complete combustion characteristics of the diesel engine. This combustion process is due to the fuel-oxidiser mixing at higher temperatures than these that would occur with a petrol engine [7]. In addition, diesel engines have less CO_2_ (carbon dioxide) and CO (carbon monoxide) emission although other pollutants such as NO (nitric oxide) and NO_2_ (nitrogen dioxide) emissions are higher due to the higher temperature resulting in more bonding between nitrogen (N) and oxygen (O_2_) atoms.

Diesel engines produce up to 100 times more particulate matter (PM) compared to petrol engines [8]. PM is measured and grouped by its aerodynamic diameter, and air pollution studies mainly concentrate on PM of 10 *μ*m (PM_10_) but diesel engines produce much smaller particles mainly with aerodynamic diameters of 2.5 *μ*m (PM_2.5_) or even ultrafine particles at the nanoscale level (<100 nm-PM_0.1_). These PMs penetrate deeper into inner regions of the human respiratory system such as the pulmonary region where they may translocate further into the cardiovascular system [9] and exert a wide range of toxicological effects. Consequently, health effects caused by diesel exhaust are of major concern.

Toxicology studies have traditionally depended on animal-based (*in vivo*) experiments, which pose a number of concerns including cross-species correlation, ethical considerations, and economic constraints. In recent years, *in vitro* toxicology methods have gained popularity and have been implemented in regulations and risk assessments [10, 11]. In addition, *in vitro* toxicology allows the usage of human cells for toxicity testing which eliminates the need for cross-species correlation and provides a method to understand toxicity mechanisms of different chemicals, although some issues on *in vitro-in vivo* correlation still remain [12]. Besides the multicellular nature and complex structure of the distal lung, the pulmonary epithelium is composed of two distinct cell types of alveolar type I and alveolar type II serving diverse essential functions in the alveolar region [13]. In this study, human pulmonary type II epithelial cells (A549) were used as a model for the human pulmonary system since the main target organ for diesel exhaust especially particulates is the pulmonary epithelium and macrophages [14]. 

To date, a number of *in vitro* based studies have investigated the toxicity of diesel exhaust [15–17]. However, most of these test methods were based on assessing pure individual components rather than the complex mixture of diesel exhaust. These methods do not allow direct exposure of cells to airborne diesel exhaust pollutants. An optimal *in vitro* exposure system for studying the cellular response following exposure to airborne pollutants needs to meet several criteria including a very close contact between target cells and test atmosphere. Recently, a number of published studies have used biphasic cell culture technique in which human cells are directly exposed to airborne pollutants on the apical side while receiving required nutrients to maintain viability at the basal side [18–20]. 

The aim of this study was to investigate the cytotoxicity of diesel exhaust using a dynamic direct exposure method developed by our research group [19, 21, 22] which exposes cells directly to airborne pollutants at the air-liquid interface as would occur in the human respiratory system. Studies were performed in a laboratory controlled environment and a field study environment to further verify the potential application of the dynamic direct exposure method for toxicity testing of diesel exhaust emissions *in vitro* and to study the toxicity of complex atmospheres generated by the combustion of diesel fuel.

## 2. Materials and Methods

### 2.1. Cell Types and Culture Conditions

Human pulmonary type II-like epithelial cells (A549, ATCC No. CCL-185) were cultured in sterile, vented 75 cm^2^ cell culture flasks with Dulbecco's modified eagle medium: Ham's F12 nutrient mixture (DMEM/F12; Gibco, USA) supplemented with 5% (v/v) newborn calf serum (NCS; Gibco, USA) and 1% (v/v) antibiotic solution containing: 10,000 units of penicillin, 10,000 *μ*g of streptomycin, and 29.2 mg of L-glutamine/mL (Gibco, USA). Cultured cells were kept at 37°C in a humidified 5% CO_2_ incubator.

Cells were grown on porous membranes (0.4 *μ*m) in Snapwell inserts. Snapwell inserts are a modified Transwell culture insert with a 12 mm diameter which provides a growth surface area of 1.12 cm^2^ (clear polyester Snapwell insert, Corning) supported by a detachable ring placed in a six-well culture plate. To seed cells, supplemented media was added to the bottom and top parts of the membrane wells (bottom: 2 mL, top: 0.5 mL) and plates incubated for 1 hr at 37°C to improve cell attachment. Confluent A549 cell layers were removed using an enzyme method (0.5% trypsin-edta; Gibco, USA), centrifuged for five mins, and resuspended in supplemented culture media. Afterwards, the supplemented media on the top part were replaced with supplemented media containing 30 × 10^4^ cells (except background control wells) and plates incubated for 24 hrs to allow cell attachment to the membrane. 

### 2.2. Direct Exposure of Target Cells to Diesel Exhaust

Cell attachment was checked using a light microscope by observing confluence (75–80%). The medium was then removed from the top part and the membrane was washed with Hank's balanced salt solution (HBSS; Gibco, USA) and then transferred into the modified dynamic exposure chambers (Harvard Apparatus, Inc., USA) for direct exposure to diesel exhaust. The bottom part of the chamber was filled with serum-free media supplemented with antibiotics (1% v/v) and maintained at 37°C using preheated blocks. The membrane was positioned at the air/liquid interface which allows cells to be exposed to air pollutants while simultaneously allowing lung cells to receive nutrients from the basolateral side. 

### 2.3. Optimal Airflow

Optimal airflow was determined by comparing cell viability of cells grown on the porous membranes. Cell viabilities were determined using the MTS assay with comparisons made between control (0 mL/min) and various airflow rates (up to 100 mL/min). Comparisons were performed using one-way ANOVA with Bonferroni's comparison test with *P* < 0.05 considered as statistically significant.

### 2.4. Generation of Diesel Exhaust

The diesel fuel used for this study was purchased from a commercial fuel station (Vortex premium diesel; CALTEX), which complied with Australian Standard 3570-1998 [23] listed in Table 1.

For the laboratory-based study, a Volvo 230 hp truck engine, running on idle mode with no load, was employed. The engine was switched on and set on idle setting for 15 mins for warming up before the exposure of target cells to diesel exhaust. Following the stabilisation period, the exhaust was delivered to the dynamic direct exposure chambers using negative pressure pumps (SKC Inc., USA) calibrated at very low flow rates (≤37.5 mL/min), and cells were exposed to diesel exhaust for 15, 30, and 60 minutes.

For the field study, a diesel engine powered passenger car (Volkswagen Passat; model 2006) was used. Similarly, before exposure of target cells to diesel exhaust, the engine was switched on and set on idle setting for 15 mins for warming up, and cells were then exposed to diesel exhaust for 15, 30, and 60 minutes. 

### 2.5. Monitoring and Analysis of Exhaust Emissions

**Figure 1:**
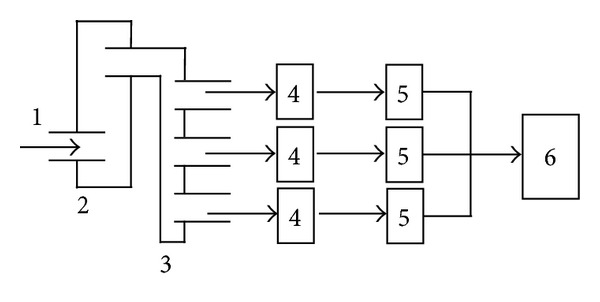
Diesel exhaust dynamic direct exposure and sampling systems including (1) sampling input port, (2) mixing chamber, (3) distribution channels, (4) exposure chambers, (5) rotameters, (6) and negative pressure pump.

During the exposure time, exhaust emissions were continuously monitored using MX6 iBrid gas monitors (Industrial Scientific, USA) capable of measuring CO, CO_2_, NO, NO_2_, O_2_, and Cl gases (Figure 1). Exhaust particulate matter was collected on quartz filters (37 mm diameter, mounted on a three-piece cassette; SKC, USA) at 2.5 L per min flow. The filters were analysed using thermal optical organic carbon/elemental carbon using the principles of the NIOSH 5040 Method to determine the exhaust's carbon content.

### 2.6. Postexposure Incubation

To investigate further any nonacute or delayed toxic effects caused by diesel exhaust and/or cell viability recovery, toxic effects of diesel exhaust in human alveolar epithelial cells were investigated after 0 and 24 hrs after incubation. 

### 2.7. Cytotoxicity Endpoints

To assess the cytotoxicity of diesel exhaust, a range of *in vitro *bioassays including MTS ([3-(4,5-Dimethylthiazol-2-yl)-5-(3-carboxymethoxyphenyl)-2-(4-sulfophenyl)-2H-tetrazolium]), NRU (neutral red uptake), and ATP (Adenosine triphosphate) assays were utilised measuring various biological endpoints, following 0 h and 24 hrs after exposure incubation.

### 2.8. MTS: Tetrazolium Salt Assay

The Promega CellTiter 96 AQ_ueous_ Non-Radioactive Cell Proliferation Assay was used to measure the toxicity of exhaust by determining the number of viable cells in culture [24]. This assay has been successfully used for the toxicity testing of air borne contaminants [25, 26]. The MTS assay works on the basis of the viable cells' ability to convert soluble tetrazolium salt into a formazan product. The MTS reagent was mixed with an electron coupling agent PMS (phenazine methosulfate; Sigma, USA) to allow faster bioreduction and faster production of the formazan product [27].

Following postexposure incubation of either 0 hrs or 24 hrs, serum-free media was replaced with fresh media supplemented with serum in both the bottom (2 mL) and top parts (0.4 mL) of the membrane. The MTS reagent was then added to the top part of the membrane (0.1 mL), and the membranes were then incubated at 37°C for 1 hr. Following the incubation period, aliquots of 100 *μ*L were transferred to a 96-well plate with 3-4 replicates and the absorbance levels were recorded at 492 nm using a multiplate reader (Multiskan Ascent, Thermo Laboratories, Finland) against controls.

### 2.9. NRU: Neutral Red Uptake

The NRU assay measures the ability of viable cells to incorporate and bind neutral red (a supravital and weakly cationic dye) which penetrates all membranes and accumulates intracellularly in lysosomes [28]. Before usage, the NRU solution was centrifuged at 1500 ×g and the supernatant was filter sterilised (0.22 *μ*m).

Following the postexposure incubation period, the media from the bottom part of the membrane were replaced with fresh media (2 mL), NRU solution was added to the top part (0.5 mL), and the plate was incubated for 3 hrs at 37°C. Following incubation, media was removed from the bottom and top parts of the membrane and fixative solution was added (0.5 mL) to the top part of the membrane for no more than 30 secs. Top and bottom parts of membrane were then immediately washed with HBSS, and afterwards, the solubilisation solution was added (0.5 mL) and the plate was shaken for 10 mins using an orbital mixer (Ratek Instruments, Australia). Aliquots of 100 *μ*L were transferred into the 96-well plate in 3-4 replicates and the absorbance was recorded at 540 nm with a microtiter plate reader (Multiskan Ascent, Thermo Laboratories, Finland) against controls.

### 2.10. ATP: Adenosine Triphosphate

The cellular ATP content was measured using the CellTiter-Glo Luminescent Cell Viability Assay. CellTiter-Glo Reagent induces cell lysis and generation of luminescence which is proportional to the amount of cellular ATP that cells contain, and the luminescence is then read using a luminometer [29].

Following the postexposure incubation period, media from the top part of the membrane were replaced with fresh media (0.25 mL) and an equal volume of CellTiter-Glo was added. The plate was then left at room temperature for 10 mins and then shaken using an orbital mixer (Ratek Instruments, Australia). Aliquots of 100 *μ*L were transferred into a 96-well opaque walled microtiter plate with 3-4 replicates and luminescence was recorded using a luminometer (Berthold Detection Systems, Germany).

### 2.11. Controls

Appropriate controls were prepared including an IC_100_ control (0% cell viability; media only), used for background and incubated at 37°C during the exposure time and an IC_0_ control (100% cell viability; cells only), used for reference and incubated at 37°C during the exposure time. The IC_0_ control was a supplementary verification that air control's cell viability has not been affected. 

In addition, an air control was also used to consider any reduction of cell viability induced by the dynamic airflow and as a reference for percentage of cell viability calculations of exposed cells [30]. Briefly, cells in air control were exposed to clean synthetic air (filtered with 0.2 *μ*m porous membrane), and the airflow was set at the same rate with the exhaust exposed cells. Similar to the exposed cells, control cells were grown on membranes and exposed to only purified air during the exposure time.

### 2.12. Statistical Analysis

Statistical analyses and graph generation were performed using GraphPad prism software. Results of the experiments were expressed as mean ± standard deviation (M ± SD). The percentage of cell viability was calculated by assuming the absorbance of IC_0_ as 100% cell viability [30] and the background absorbance at IC_100_ (media only). One-way ANOVA with Bonferroni multiple comparisons was used to compare and determine the significance of the difference between the average cell viability of control cells and exposed cells. Differences were considered statistically significant at *P* < 0.05.

## 3. Results

### 3.1. Optimal Control Airflow Rate

**Figure 2:**
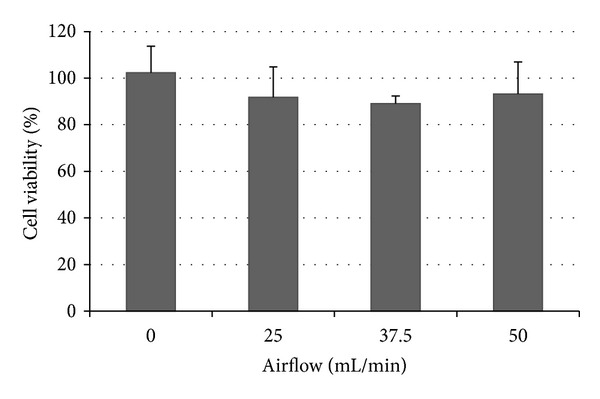
Effect of airflow on cell viability after 15 minutes of exposure to synthetic clean air. Each point is expressed as M ± SD.

Various air flow rates were tested to determine the optimal air flow rate to deliver the maximum contaminants yet not affecting cell viabilities. Cell viability was determined using the MTS assay and comparison between control (0 mL/min) and other flow rates was performed using one-way ANOVA with Bonferroni's comparison test as shown in Figure 2. It was determined that sampling flow less and including 50 mL/min did not produce any statistically significant reduction in cell viability.

### 3.2. Results: Laboratory-Based Study

**Figure 3:**
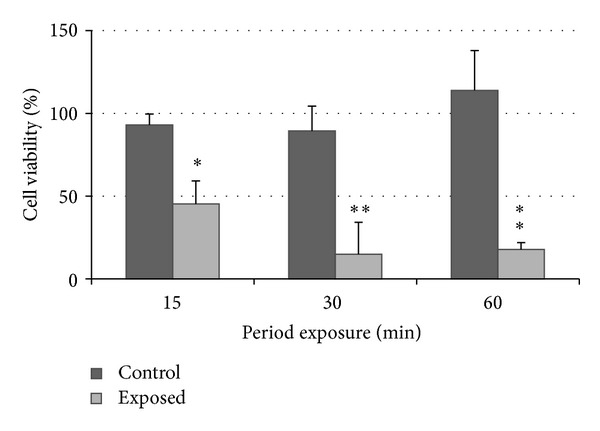
A549 cell viability following exposure to diesel exhaust generated in the laboratory-based study between 0 to 60 mins at 37.5 mL/min flow, as measured by the ATP assay; ∗ denotes *P* < 0.05 and ∗∗ denotes *P* < 0.01.

**Figure 4:**
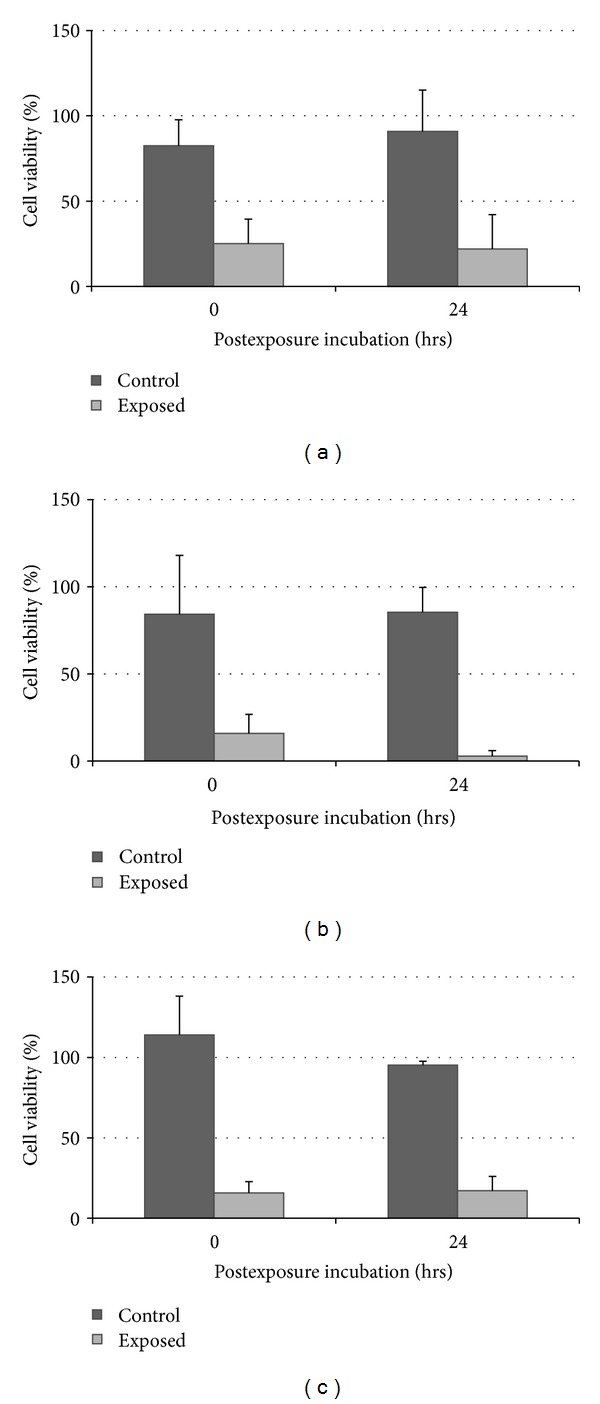
Comparison of A549 cell viability with 0 and 24 hrs postexposure incubation periods following exposure to diesel exhaust in laboratory-based studies at 37.5 mL/min flow with (a) MTS assay, (b) NRU assay, and (c) ATP assay.

The results are presented in Figures 3 and 4. Cell viabilities were expressed as a percentage of control as M ± SD, and each data point represented an average of 3 different repetitions at each exposure period. Three different *in vitro* assays (ATP, MTS, and NRU) were performed, comparison of results was made with ANOVA and Bonferroni's comparison, and differences were considered statistically significant at *P* < 0.05.

Figure 3 showed a statistically significant reduction in cell viability (determined with the ATP assay) following exposure to diesel exhaust at 37.5 mL/min flow between 15 and 60 mins exposure. Figure 4 showed no statistically significant reduction in cell viabilities following the longer postexposure incubation which suggested that longer postexposure caused no further statistically significant reduction in cell viability.

### 3.3. Results: Field-Based Study

**Figure 5:**
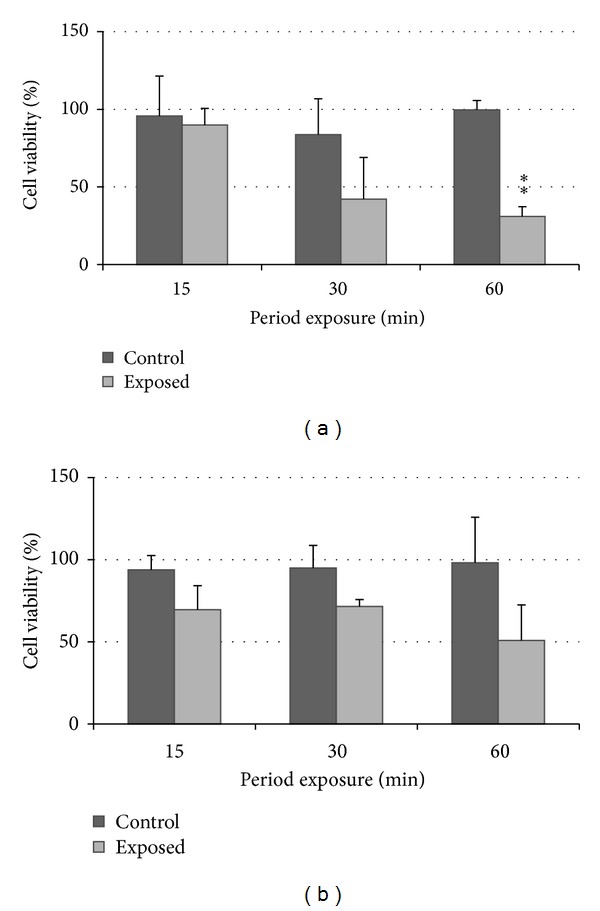
A549 cell viability following exposure to diesel exhaust generated in field-based study between 0 and 60 mins at 37.5 mL/min flow as measured by (a) ATP assay and (b) MTS assay.

**Figure 6:**
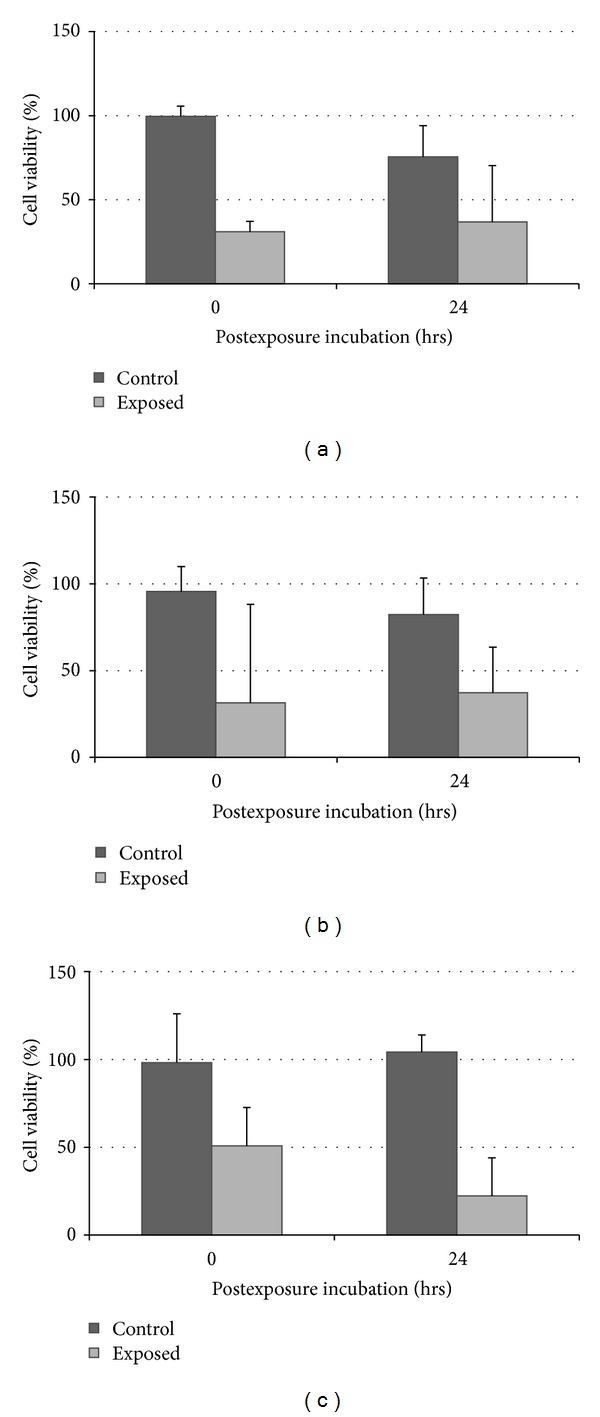
Comparison of A549 cell viability with 0 and 24 hrs postexposure incubation periods following exposure to diesel exhaust in the field-based study at 37.5 mL/min flow with (a) MTS assay, (b) NRU assay, and (c) ATP assay.

The results are presented in Figures 5 and 6. Cell viabilities were expressed as a percentage of control as M ± SD, and each data point represented an average of 3 different repetitions at each exposure period. Three different *in vitro* assays (ATP, MTS, and NRU) were performed, comparison of results was also performed with ANOVA and Bonferroni's statistical tests. Differences were considered statistically significant at *P* < 0.05.

Figure 5 showed a statistically significant reduction in cell viability (determined with the ATP assay) following exposure to diesel exhaust at 37.5 mL/min flow at 60 mins exposure.  Figure 6 showed no statistically significant reduction in cell viabilities following longer postexposure incubation which suggested that the longer postexposure caused no further statistically significant reduction in cell viability similar to the laboratory-based experiment.

### 3.4. Diesel Exhaust Gas Monitoring

**Figure 7:**
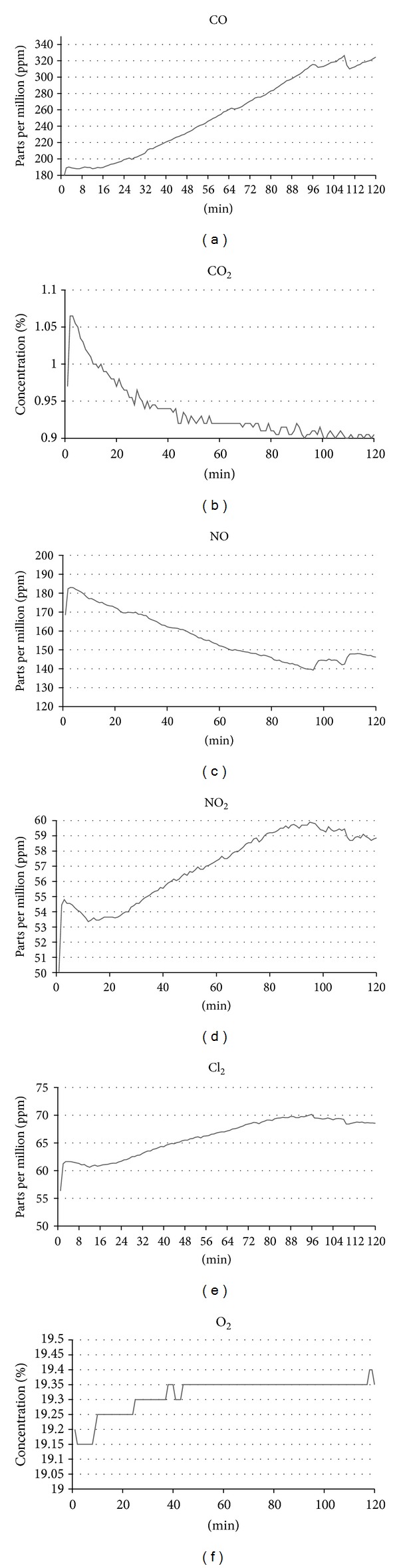
Gas monitoring of the laboratory-based engine exhaust up to 2 hrs following initial warm-up period for (a) CO; (b) CO_2_; (c) NO; (d) NO_2_; (e) Cl_2_; (f) O_2_.

**Figure 8:**
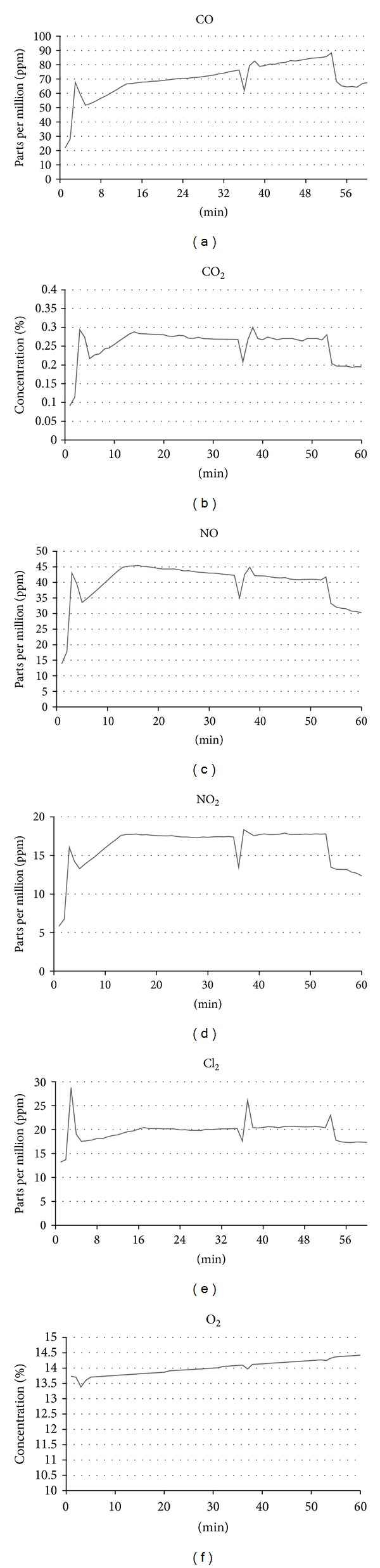
Gas reading monitoring of the field-based engine exhaust up to 2 hrs following initial warm-up period: (a) CO; (b) CO_2_; (c) NO; (d) NO_2_; (e) Cl_2_; (f) O_2_.

The level of gaseous emissions from diesel engines was also monitored and recorded to determine the concentration of gases during the exposure periods. Gases monitored included CO, CO_2_, NO, NO_2_, Cl_2_, and O_2_. Gas readings were measured twice and the average results were plotted in Figure 7 for the laboratory-based study and Figure 8 for the field-based study.

### 3.5. Organic and Elemental Carbon Analysis of Exhaust

The organic and elemental carbon content analysis for the laboratory-based engine and field-based engine exhausts is displayed in Tables 2 and 3, respectively.

## 4. Discussion

The cytotoxicity of diesel exhaust was investigated using the dynamic direct exposure method in which human lung cells were grown on a porous air/liquid membrane allowing simultaneous exposure of cells to exhaust whilst receiving nutrients. A laboratory-based diesel engine and field-based diesel powered car were used to generate diesel exhaust which was delivered to human target cells through a dynamic exposure chamber employing a horizontal diffusion system. Cytotoxicity of the diesel exhaust was investigated in A549 human pulmonary type II like epithelial cells using the MTS, NRU, and ATP *in vitro* cytotoxicity assays. 

Diesel particulate organic and elemental carbon components were analysed using the NIOSH 5040 method. A higher elemental carbon content indicates a higher amount of particulates within the exhaust whereas the organic carbon content indicates the amount of molecules with carbon in its chain [31]. The results in Table 2 showed the expected increase in total carbon corresponding with the longer exposure period, and the laboratory-based experiment showed higher elemental carbon component content at the longer sampling period. This change of ratio became evident following the 30–60 mins sampling period, which suggests that the laboratory engine at idle setting reached equilibrium state only following 30–60 mins. With the field engine, the ratio had no significant change suggesting that the diesel particulate filter effectively reduced emission of elemental carbons consisting of PMs.

The results of both laboratory and field studies suggest that the exposure of A549 lung cells to diesel exhausts for a period of 60 mins caused cellular injury (Figures 3 and 5). However, cells exposed to control air remain viable for up to 1 hr, which supports the research by Aufderheide et al. (2003) and Bakand et al. (2007) where cells were exposed to clean air at low flow rates up to 2 hrs without any significant cell viability reduction [26, 32, 33]. In other studies, A549 cells grown on membranes were exposed to diesel, petrol exhausts [34, 35], and smoke from polymer combustion [22]. Cheng et al. investigated production of IL-8 with the exposure period up to 6 hrs, and Tsukue et al. investigated cell viability and gene expression after one hour exposure period, while Lestari et al. investigated cell viability after 30 minutes exposure to polymer combustion smoke [36]. Recently, spectroscopy and atomic force microscopes were used complementary to ELISA to observe additional biophysical effects on cells [37].

For both field- and laboratory-based studies the NRU assay result detected most reduction in cell viability then followed by ATP and MTS assays. However, the MTS assay showed more consistent results shown by its lower SD, while the NRU assay had the largest SD especially with 0 hr postexposure incubation. This high SD could be caused by the formation of crystals as observed in a study by Husoy et al. (1993) [38] although in this study steps were taken to minimise the crystal formation by incubating the NR medium overnight, centrifuging and filtering it using a 0.22 *μ*m pore filter as performed by Borenfreund and Puerner (1985) [28].

Following exposure to diesel exhaust, the cells were either immediately used for *in vitro* assays or incubated for a further 24 hrs to determine any delayed cytotoxic effects. It is suggested that cell functions such as cell-division capability and delayed-onset toxicity may only be obvious following further incubation [39]. In comparison to results in Figures 4 and 6, there is no further statistically significant reduction in cell viability hence indicating lack of recovery. However, both 0 and 24 hrs postexposure incubations can be considered depending on the kind of response observed (immediate versus delayed), in which longer postexposure incubation reflects a more diverse range of responses from cells following exposure to diesel exhaust. In addition, both the NRU and MTS assays showed similar decreases in cell viability following 24 hrs postexposure incubation which demonstrated the correlation of results between the two assays in determining basal cytotoxicity of pollutants [40, 41].

This study investigated time-dependent toxicity of diesel exhaust by setting the diesel engine at idle setting and exposing human lung target cells to the exhaust for a range of exposure periods including 15, 30, and 60 mins. However, as can be seen in Figures 7 and 8, NO emission decreased while NO_2_ increased which is caused by an increase in the temperature of the combustion environments (monitored by its coolant temperature), while CO emission is increased. This trend of increasing NO_2_ ratio is usually seen in diesel engines equipped with diesel particulate filters and running at higher loads [42, 43]. Since this study uses idle settings, the engine temperature may have affected the ratio change. These various gaseous trends make it difficult to maintain a constant generation of exhaust; hence, a dose-dependent toxicity study may have more reproducible exhaust in which the amount of pollutant will be increased by increasing load or rpm of the engine [44]. 

Future works will concentrate on assessing the toxicity of diesel exhaust on various cells to determine toxicity on specific organs. One possible organ of interest is the liver, since the ultrafine particulates may translocate from alveoli into the brain and ultimately into the bloodstream to the liver [9, 45] where the health risks are not well-known. In addition, specific components of diesel exhaust such as PM need to be investigated further as these nanosized particles have major human health impacts especially on the respiratory system [46–48]. In addition, multiple assays could be used in studies to provide more data to elucidate the toxicity mechanisms of diesel exhaust. Other human target cells can be used to represent another organ of toxicological significance such as the liver via the hepG2 cell line or a specific primary culture could be used to assess a greater range of toxic responses and may reduce the necessity of *in vivo* systems [49] and other laborious systems with specific cells [50–52]. It is preferable that the same culture media are used as different culture media might affect *in vitro* cytotoxicity assays results such as in IL-6 secretion [53].

In conclusion, the obtained results suggested that the dynamic direct exposure method, in parallel with real-time air monitoring techniques, has the potential to be implemented for both laboratory- and field-based toxicity studies of diesel exhaust *in vitro*. The main advantage of this method is its closer approximation to real-life situations in which cells continuously receive nutrients whilst being exposed to air pollutants.

## Figures and Tables

**Table 1:** Australian Standard 3570 on diesel fuel specification.

	CALTEX Vortex premium diesel	AS3570 requirement
Density at 15°C	0.82–0.87 kg/L	0.83 kg/L
Minimum cetane number	50	45
Maximum sulphur content (by mass)	0.5%	<10 ppm
Energy density	N/A	35.9 megajoules/litre or 43.3 megajoules/kg

**Table 2:** Elemental carbon (EC), organic carbon (OC), and total carbon (TC) content of laboratory based engine.

Sampling period (mins)	EC concentration (*µ*g/m^3^)	OC concentration (*µ*g/m^3^)	TC concentration (*µ*g/m^3^)	EC/OC ratio	EC/TC ratio
15	3.639	61.225	64.863	0.059	0.056
30	2.611	37.455	40.066	0.070	0.065
60	1.133	10.981	12.114	0.103	0.094

**Table 3:** Elemental carbon (EC), organic carbon (OC), and total carbon (TC) content of field based engine.

Sampling period (mins)	EC concentration (*µ*g/m^3^)	OC concentration (*µ*g/m^3^)	TC concentration (*µ*g/m^3^)	EC/OC ratio	EC/TC ratio
30	2.379	0.863	3.241	2.758	0.734
60	2.110	0.858	2.968	2.460	0.711

## Abbreviations

ATP: Adenosine triphosphate
A549: Human pulmonary type II epithelial cells
ATCC: American type culture collection
DMEM/F12: Dulbecco's modified eagle medium: Ham's F-12 nutrient mixture
HBSS: Hank's balanced salt solution
MTS: [3-(4,5-Dimethylthiazol-2-yl)-5-(3-carboxymethoxyphenyl)-2-(4-sulfophenyl)-2H-tetrazolium salt] assay
NRU: Neutral red uptake
PMS: Phenazine methosulfate.
